# Comparison of Manual Two-dimensional and Automated Three-dimensional Methods of Assessing Shoulder Joint Morphology through Computed Tomography Images

**DOI:** 10.1055/s-0044-1786821

**Published:** 2024-07-08

**Authors:** Geraldo da Rocha Motta Filho, Marcus Vinícius Amaral, Márcio Cohen, Marcio Schiefer de Sá Carvalho, Raphael Soares da Fonseca, Ana Carolina Leal de Oliveira

**Affiliations:** 1 Centro de Cirurgia de Ombro e Cotovelo, Instituto Nacional de Traumatologia e Ortopedia, Rio de Janeiro, RJ, Brasil; 2 Divisão de Traumatologia e Ortopedia (DITRO), Instituto Nacional de Traumatologia e Ortopedia, Rio de Janeiro, RJ, Brasil; 3 Divisão de Ensino e Pesquisa, Instituto Nacional de Traumatologia e Ortopedia, Rio de Janeiro, RJ, Brasil

## Abstract

**Objective** To evaluate the interobserver agreement in the measurement of anatomical parameters of the shoulder using manual methods of two-dimensional (2D) computed tomography (CT) unformatted in the plane of the scapula and to compare them with the automated measurement obtained through the Blueprint (Wright Medical, Memphis, TN, United States) software, which uses reconstructed three-dimensional (3D) images.

**Methods** The present is a cross-sectional study in which 2D CT images of 38 patients with different diagnoses were used. The anatomical parameters were measured by the manual methods described by Friedman et al., the glenoid vault method, the Maurer et al. method, and shoulder subluxation according to Walch et al., by five independent qualified surgeons and compared with the parameters obtained through the Blueprint automated software.

**Results** Significant differences were found between the manual measurement version obtained through the Friedman et al. method and the automated version. The mean values found for inclination did not show statistically significant differences among the methods. The mean value found for subluxation showed significant differences between the average observed in the analyses performed by the automated method and those performed by the surgeons.

**Conclusion** The manual measurements of glenoid version and inclination performed by experienced surgeons are effective, and the vault method is superior to the Friedman et al. method in the analysis of severe glenoid deformities.

## Introduction

Correction of joint deformities in the glenoid is essential to perform shoulder arthroplasty, and it can directly impact the functional result and survival of the implant. 1 Therefore, preoperative planning is essential for the successful performance of total shoulder arthroplasty. 1 2 3 4

Traditionally, the assessment of glenoid morphology is performed using angular measurements on two-dimensional (2D) computed tomography (CT) images, which have demonstrated low accuracy and inter- and intraobserver agreements. 5 The use of three-dimensional (3D) CT images has shown limited improvements as the reconstruction of the scapula plane requires reformatting and processing of 2D images. 6 7

Thus, the emergence of three-dimensional (3D) CT images associated with the development of automated computer software is intended to control the limitations and inaccuracies of manual methods and the reformatting of CT slices in the scapular plane. 8 9 10 11

Therefore, the aim of the present study was to evaluate the interobserver agreement in the measurement of anatomical parameters of the shoulder using manual methods from 2D CT unformatted in the plane of the scapula and to compare them with automated measurement that uses reconstructed 3D images.

## Materials and Methods

### Selection of Cases

After approval by the institutional Ethics in Research Committee (under no. 35243920.4.0000.5273), a study was carried out to evaluate the CT images of the shoulder joint in patients of both sexes, older than 18 years of age, diagnosed with osteoarthritis.

In total, 38 CT scans were randomly selected from exams performed for patients at the institution from January 2015 to December 2019. All CT exams were performed using the same device (model Brilliance, Philips, Amsterdam, Netherlands), with 64 channels, with the patient in the supine position.

The inclusion criteria were CT scans that had triplanar, coronal, sagittal, and axial sections, with a minimum section thickness of 1 mm, in which the entire scapula was visualized and were processed by the automated surgical planning software selected for the experiment. Images of patients who had undergone previous surgery in the shoulder and who had artifacts on CT images were excluded, such as the presence of a metallic implant or other anatomical changes that could affect the segmental processing by the automated software.

### Manual Measurement

**Fig. 1:**
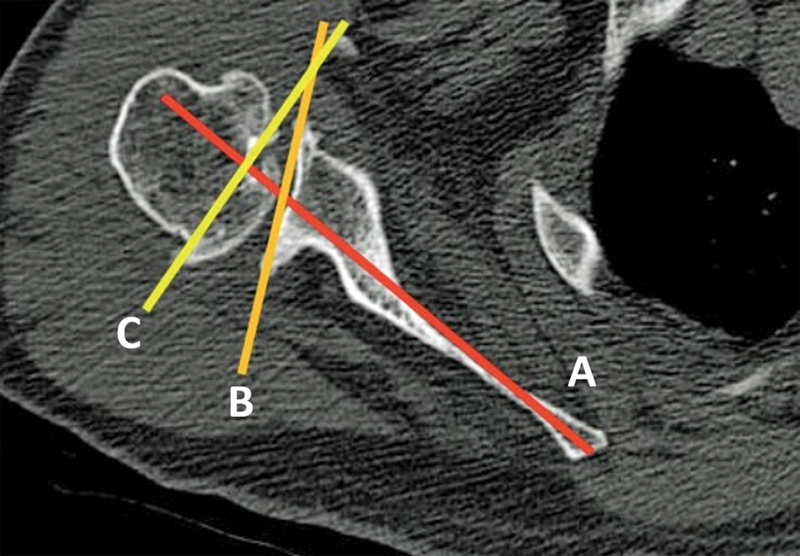
Friedman et al. 12 method to evaluate the glenoid version. We used the fourth cut distal to the last cut in which the tip of the coracoid process was visualized in the axial plane. The glenoid version was determined by the angle, as shown: ( **A** ) Friedman et al. 12 line, ( **B** ) glenoid line, and ( **C** ) line perpendicular to the scapular axis. A positive value of the glenoid version angle was interpreted as anteversion, while a negative value was interpreted as retroversion.

**Fig. 2:**
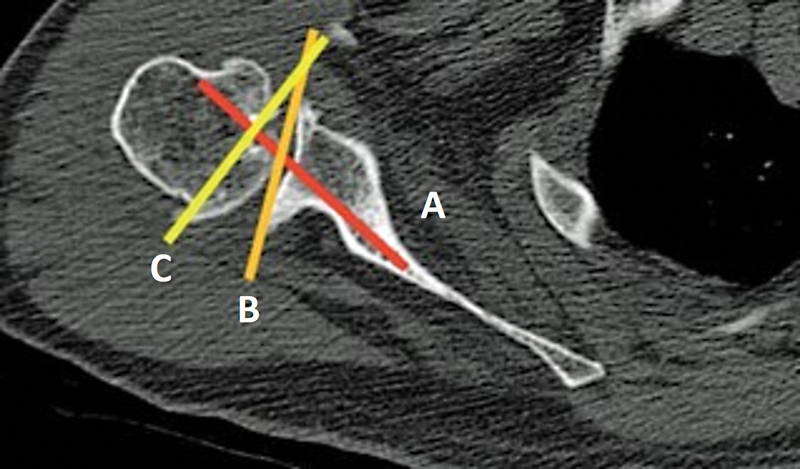
The vault method for the measurement of the version, which was defined in the axial section as a triangle composed of the anterior and posterior walls of the scapula neck and the glenoid articular surface. The angle is determined by the lines: ( **A** ) vault axis, ( **B** ) glenoid line, and ( **C** ) line perpendicular to the vault axis.

**Fig. 3:**
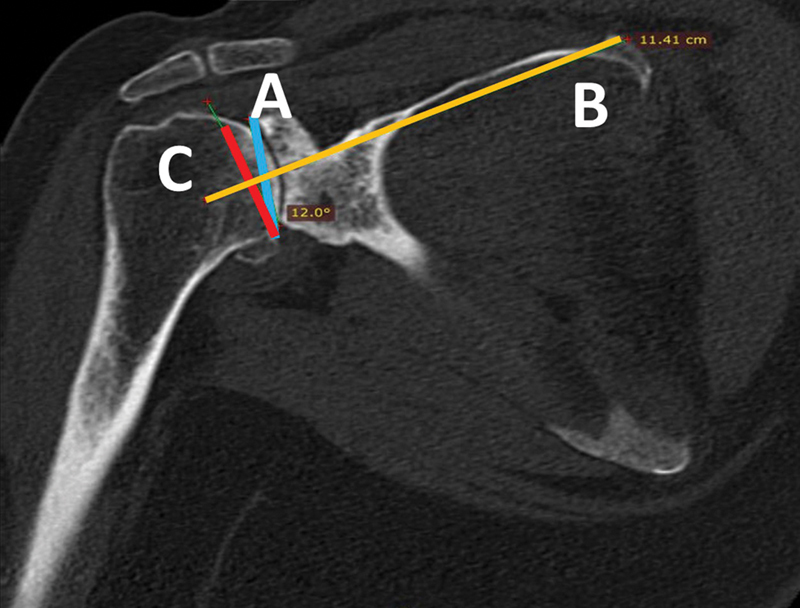
Maurer et al. 14 method for the measurement of the glenoid inclination, which was performed in the oblique coronal plane of the computed tomography scan, and the slice that best captured the floor of the supraspinatus fossa was selected. ( **A** ) Line tangent to the anterior and posterior edges of the glenoid, ( **B** ) supraspinatus fossa line, and ( **C** ) line perpendicular to line B determining the glenoid inclination.

**Fig. 4:**
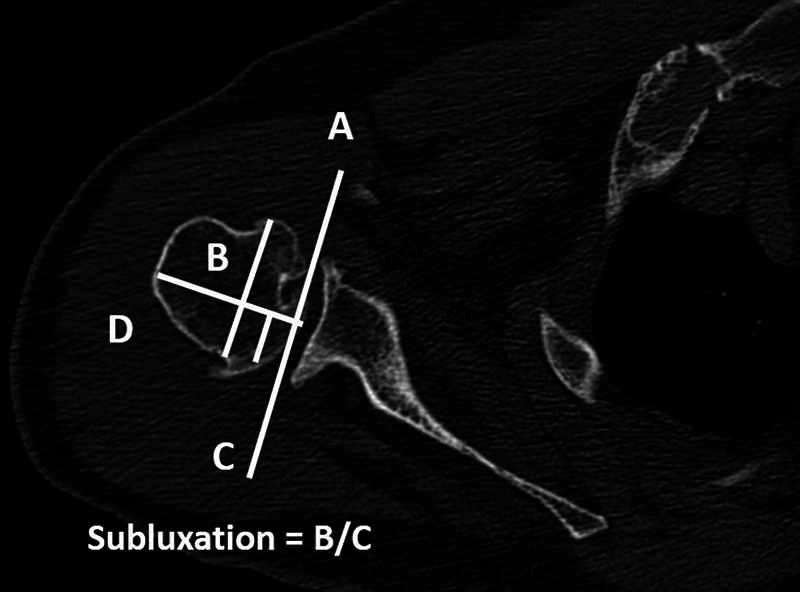
Walch et al. 4 method to determine humeral head subluxation. ( **A** ) Line tangent to the anterior and posterior edges of the glenoid, ( **B** ) line perpendicular to the face of the glenoid at its midpoint, ( **C** ) line parallel to line A, dividing the middle third of the humeral head, and ( **D** ) part of the humeral head posterior to the center of the glenoid. Subluxation index = D/C.

The 2D CT images that were not formatted in the scapula plane were used for the manual measurement of the version, according to the Friedman et al. 12 ( Fig. 1 ) and glenoid vault methods, described by Matsumara et al. 13 ( Fig. 2 ), and of the glenoid inclination using the Maurer et al. 14 ( Fig. 3 ) method in addition to the measurement of the percentage of humeral head subluxation according to Walch et al. 4 ( Fig. 4 ).

These measurements were performed independently by five orthopedists specialized in shoulder surgery using the RadiAnt DICOM Viewer software (Medixant, Poznan, Poland). 15 The observers were uniformly instructed, as described below, to standardize the measurements. All observers were blinded to each other's results.

### Automated Measurement

**Fig. 5:**
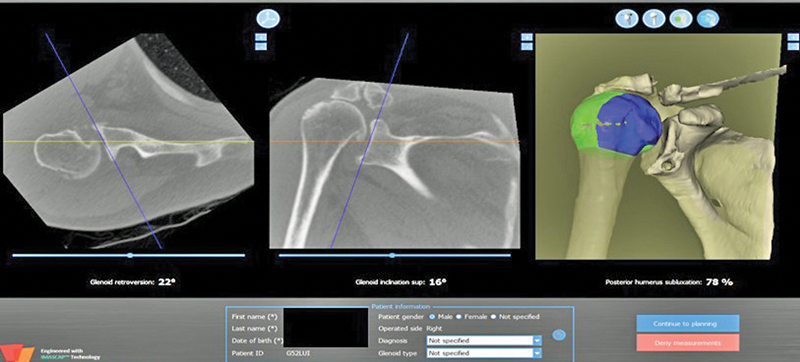
Initial screen of the Blueprint software showing the values of the glenoid version and inclination, and humeral head subluxation.

The automated measurement used the CT images in the Digital Imaging and Communications in Medicine (DICOM) format that were processed by the Blueprint 9 (Wright Medical, Memphis, TN, United States) software. The software performs an automatic segmentation process, determines the scapula and glenoid planes, and then performs measurements, providing the version and inclination values, in addition to the percentage of subluxation of the humeral head ( Fig. 5 ).

### Statistical Analysis

The interclass correlation coefficient (ICC) was calculated to determine the variability in the manual measurements among surgeons.

For the descriptive statistics, data were presented as mean, standard deviation, maximum, and minimum values. The comparison of the measurements obtained by the different methods of evaluating the version was performed using the Friedman et al. 12 test, followed by the Dunn posttest. The comparison between the measurements of inclination and subluxation by manual and automated methods was performed using the Wilcoxon test. The cases were categorized according to the severity of the version and inclination, using intervals of 0 to 10°, and > 10° when indicated. Those intervals were chosen based on studies that have shown that the normal glenoid version is close to 0°, sometimes with slight anteversion but more often slight retroversion with values typically lower than 10° in either direction, 16 and that the normal intrinsic glenoid inclination angle is generally between 0 and 10°. 17 All analyses were performed using the IBM SPSS Statistics for Windows (IBM Corp., Armonk, NY, United States) software, version 21.0.

## Results

The present study included 38 cases, 17 of which were diagnosed with cuff tear arthropathy, and 21, with osteoarthritis.

The ICC for the manual measurements is described in Table 1 . The mean version values obtained by the Friedman et al., 12 vault, and automated methods are presented in Table 2 . The vault method was similar to the automated method ( *p* > 0.99), while the Friedman et al. 12 method tended to underestimate this measure, both in relation to the automated and vault methods ( *p* = 0.003) ( Table 2 ).

**Table 1:** Interclass correlation coefficients for version, by the Friedman et al. 12 and vault methods, inclination, and subluxation

Variables	ICC	Inferior confidence interval	Superior confidence interval
**Version according to the Friedman et al.** 12 **method**	0.847	0.751	0.913
**Version according to the vault method**	0.845	0.744	0.931
**Inclination**	0.890	0.821	0.938
**Subluxation**	0.840	0.712	0.917

**Table 2:** Version measurement

	Blueprint	Friedman et al. 12	Vault
**Version (mean ± standard deviation)**	10.45° ± 7.1° ^a^	8.07° ± 6.1° ^a, b^	10.42° ± 6.2° ^b^
**Average difference** **(95% confidence interval)**	–	2.37° (0.79–3.9°)	0.023° (−1.8–1.9°)
**Maximum difference**		11°	13.64°

Notes: ^a^ *p* = 0.0059, Friedman et al. 12 test, Dunn posttest; ^b^ *p* = 0.0005, Friedman et al. 12 test, Dunn posttest.

The differences between the version measurements made by Blueprint and the Friedman et al. 12 method were lower than 5° in 23 cases (60.5%), between 5 and 10° in 14 cases (36.8%), and greater than 10° in 1 case (2.6%). Such proportions were similar to those found in the comparison between Blueprint and the vault method: 22 cases (57.8%), 13 cases (34.2%), and 3 cases (7.8%), respectively ( *p* = 0.57) ( Table 3 ).

**Table 3:** Number of cases in which the mean obtained by measurements using manual methods differed from automated measurements, stratified by degrees of difference

	Friedman et al. 12	Vault
**Difference < 5° in relation to the Blueprint software: n (%)**	23 (60.5%)	21 (57.8%)
**Difference of 5–10° in relation** **to the Blueprint software: n (%)**	14 (36.8%)	13 (34.2%)
**Difference > 10° in relation to** **the Blueprint software: n (%)**	1 (2.6%)	3 (7.8%)

Notes: Chi-squared test; *p* = 0.57.

Inclination showed no statistical difference between the automated and manual methods (11.85° ± 9.8° versus 11.24° ± 5.44°; *p* = 0.377) ( Table 4 ). Subluxation measurements resulted in higher values in the automated method in comparison to the manual one (60.08° ± 14.72° versus 48.47° ± 7.67°; *p* < 0.0001) ( Table 4 ).

**Table 4:** Measurement of inclination and subluxation

	Blueprint	Manual	*p*
**Inclination (mean ± standard deviation)**	11.85° ± 9.8°	11.24° ± 5.44°	0.377
**Subluxation (mean ± standard deviation)**	60.08° ± 14.72°	48.47° ± 7.67°	< 0.0001

Note: Wilcoxon Test.

Cases were categorized considering those presenting a glenoid inclination within the normal range (−10 to +10°) and those with anatomical variation of this parameter. For cases within the normal range, the vault method resulted in significantly higher values than the automated ( *p* = 0.04) and the Friedman et al. 12 methods ( *p* = 0.0004). Interestingly, for cases with a version higher than 10°, the mean version obtained with the automated method was significantly higher than that obtained with Friedman et al. 12 method ( *p* = 0,001), but similar to that obtained with vault method ( *p* = 0.13). The mean values for subluxation obtained with the automated method were significantly higher than those obtained with the manual method in both subgroups evaluated ( Table 5 ).

**Table 5:** Comparison regarding the mean version, inclination, and subluxation using the automated and manual methods, categorized by version measured by the automated method

Version (°)		Parameter		*p* -value
**Version (°): mean ± standard deviation**				
	Blueprint	Friedman et al. 12	Vault	
0–10 (n = 21)	5.6 ± 2.8 ^a^	5.4 ± 4.0 ^b^	7.7 ± 3.9 ^a, b^	0.01*
> 10 (n = 17)	16.3 ± 6.5 ^c^	11.3 ± 6.9 ^c^	13.7 ± 7.0	0.0061*
**Inclination (°): mean ± standard deviation**				
	Blueprint	Manual		
0–10 (n = 21)	11.5 ± 11.5	10.9 ± 6.0		0.7 ^#^
> 10 (n = 17)	10.8 ± 5.0	11.4 ± 4.8		0.5 ^#^
**Subluxation (°): mean ± standard deviation**				
	Blueprint	Manual		
0–10 (n = 21)	51.3 ± 10.4	45.7 ± 7.2		0.005 ^#^
> 10 (n = 17)	69.8 ± 12.5	52.0 ± 7.1		< 0.0001 ^#^

Notes: *One-way analysis of variance (ANOVA); ^a^ *p* = 0.04; ^b^ *p* = 0.0004; ^c^ *p* = 0.001; Tukey multiple comparisons test; ^#^ *t* -test.

The cases were then categorized considering inclination, measured by the automated method. For cases with inclination within the normal range, the mean version obtained with the vault method was significantly higher than that obtained with the Friedman et al. 12 method ( *p* = 0.1), with no differences between the automated and the manual methods. For cases with inclination higher than 10°, the mean version was significantly underestimated by the Friedman et al. 12 in comparison to the automated ( *p* = 0.002) and the vault methods ( *p* = 0.007).

In cases within the normal range of inclination, this parameter was overestimated by the manual method in comparison to the automated one ( *p* = 0.001). Regarding subluxation, the mean values obtained with automated method were significantly higher than those obtained with the manual method in both subgroups evaluated ( Table 6 ).

**Table 6:** Comparison regarding the mean of the inclination using the automated and manual methods, categorized by the severity of the inclination measured by the automated method

Inclination (°)		Parameter		*p* -value
**Version (°): mean ± standard deviation**				
	Blueprint	Friedman et al. 12	Vault	
0–10 (n = 21)	8.9 ± 6.4	8.3 ± 6.2 ^a^	10.9 ± 7.3 ^a^	0.03*
> 10 (n = 17)	12.3 ± 7.8 ^b^	7.7 ± 6.3 ^b,c^	9.7 ± 4.8 ^c^	0.003*
**Inclination (°): mean ± standard deviation**				
	Blueprint		Manual	
0–10 (n = 21)	5.9 ± 2.9		8.7 ± 3.8	0.001 ^#^
> 10 (n = 17)	17.6 ± 9.9		14.0 ± 5.8	0.1 ^#^
**Subluxation (°): mean ± standard deviation**				
	Blueprint		Manual	
0–10 (n = 21)	60.33 ± 15.0		49.9 ± 6.8	0.0001 ^#^
>10 (n = 17)	58.7 ± 14.5		46.8 ± 8.6	0.000 ^#^

Notes: *One-way analysis of variance (ANOVA); ^a^ *p* = 0.01; ^b^ *p* = 0.002; ^c^ *p* = 0.007; Tukey multiple comparisons test; ^#^ *t* -test.

## Discussion

The success of shoulder arthroplasty depends on the proper positioning of the implant. 1 The surgeon's ability to identify morphological changes in the glenoid, especially regarding the version and inclination, is extremely important, as it avoids misplacements that compromise the survival of the procedure. 2 4 7

However, manual 2D methods to define glenoid morphology present limitations and inaccuracies because of the difficulty in segmenting CT images in the anatomical plane of the scapula. The discrepancies motivated the development of automated programs for preoperative planning, whose use has become increasingly frequent in an effort to improve the understanding of the anatomy and the subsequent positioning and fixation of the prosthetic components. 8 9 However, its routine use still does not occur in the practice of most surgeons who perform shoulder arthroplasties. 8 18 19 20 Moreover, there are controversies regarding the accuracy of these methods and the ideal placement of the implants. 21 22 23 24 25

In the clinical practice at low- and middle-income countries, 2D CT images unformatted in the plane of the scapula are broadly used, which, despite representing a limitation, is a reality. In the present study, manual measurements of the version, by both the Friedman et al. 12 and vault methods, as well as inclination and subluxation, showed a good ICC. Interestingly, other studies 5 6 9 10 have shown that the version of the glenoid measured on 2D CT images presents significant interobserver variability due to the variation in the coronal and sagittal rotation of the scapula in relation to the patient's position on the examination table. However, in the present study, we obtained a good ICC, which we attribute to the fact that all evaluators are shoulder surgeons with more than five years of training and with experience in performing shoulder arthroplasties.

In the present study, the mean glenoid version measured through the automated method was similar to that obtained through the vault method. However, the version measured by the Friedman et al. 12 method was lower than that obtained by the automated and vault methods. Still, based on the comparisons of the versions, only 1 case showed a difference greater than 10° when comparing the Friedman et al. 12 method with the automated method. Therefore, it was shown that the Friedman et al. 12 method is less accurate than the vault method when the automated method was used as a standard. In 2014, Matsumara et al. 13 compared the measurement of the version by the Friedman et al. 12 and vault methods and, similarly, stated that both methods present good interobserver agreement, but that the vault method enables an easier measurement, since it does not depend on the anatomical variations of the body of the scapula or its inclusion in the exam. 11 13 15 23

Remarkably, using the automated method as a standard, in cases in which the version was within the normal range, we did not observe significative differences between the automated and Friedman et al. 12 methods; however, in cases in which the version was out of the normal range (> 10°), the mean version obtained by the Friedman et al. 12 method was significantly lower than that obtained by the automated method. Altogether, these results enable us to conclude that the use of version measurement by the automated method is more important in cases in which deformities on the glenoid surface are more severe, possibly influencing the clinical practice, both in the correction of the version and in the correct positioning of the implant. This finding is compatible with that found in the literature, in which Chalmers et al. 26 demonstrated that, in deformities classified as B2 using the morphological classification proposed by Walch et al., 4 the measurement of the version by CT images is superior. 26 27

In a recent study, Reid et al. 28 found results similar to ours, showing that the measurement of the version in 2D CT scans, using the Friedman et al. 12 method, presents both high intra- and interclass correlation rates. On the other hand, this study 28 also found significant differences between measurements using manual and automated methods. However, when the cases were categorized according to version severity, the authors 28 did not find differences between the methods in the different subgroups evaluated, suggesting that the version magnitude does not influence the differences between the methods. This result contrasts with the findings of the present study, as we found that the manual method underestimated glenoid inclination, in comparison to automated method, in cases with a version greater than 10°. The different version ranges used to stratify the cases and the different diagnosis included in our series may explain these differences.

Glenoid inclination presented similar values when assessed by the manual or automated methods. Iannotti et al., 21 regarding inclination measurements on formatted CT scans, observed that there were no differences from those obtained with the automated method. In relation to unformatted images, an expressive difference was found. In 94% of the cases, the difference in inclination between the unformatted and the formatted images was greater than 5°, again a value with significance in the clinical practice. 21 In our series, when the cases were categorized according to severity of the inclination, differences were found in the measurement of mild cases (0–10° of inclination), in which the manual method resulted in a mean that was significantly higher than that obtained by the automated method ( *p* = 0.001). In a study published in 2020, Choi et al. 29 compared inclination measured by automated and 2D methods and found that the 3D method resulted in measurements that were significatively lower than those obtained with the 2D method. Although the authors 29 did not perform a subgroup analysis, the data evaluated presented mean values for version and inclination compatible with the cases included in the subgroup of mild cases of the present study.

In relation to subluxation, significant differences were found between the manual and automated methods. Other studies 30 have already reported an error intrinsic to the measurement of subluxation using the Walch et al. 4 method. The subluxation index, described by Walch et al., 4 is measured on a CT transverse cut. The scapular body has an inclination compared to the body of the patient in whom the CT cross-sections are aligned, which leads to errors in the 2D CT measurements. 5 10 11 26 27 30 Jacxsens et al. 30 concluded that measurements based on shoulders reconstructed in 3D seem to be more appropriate, as they reconstruct the bone anatomy regardless of the patient's orientation at the time of the exam, and could be, therefore, another more reliable measurement than the 2D one, which is underestimated.

An interesting finding of the present study was that the differences between the subluxation measurements obtained by the automated and manual methods tended to increase with the severity of the version, suggesting that the greater the version angle, the more the manual method underestimates the measurement of the subluxation, when the automated method is used as the default. Further studies are needed to assess the clinical impact of such a finding, once it can directly impact on surgical planning.

Chalmers et al. 26 evaluated the glenoid version and inclination and humeral head subluxation values using corrected and uncorrected CT scans, and compared these measurements with the values provided by the Blueprint software. They 26 concluded that the orientation of the slices of CT scans formatted in the plane of the scapula presented a decrease in retroversion measurements compared to unformatted CT scans. On the other hand, the authors 26 verified that there are no differences between unformatted CT scans and the values of automated programs. In 48% of the cases, the difference between the uncorrected and corrected versions was greater than 5°, which is considered significant in the clinical practice. 21 These findings are similar to the results obtained in our series.

Shukla et al. 23 used the same automated program used in the present study and found similar results between the manual and automated methods for the measurements of version and inclination, but with an important difference in the percentage measurements of subluxation of the humeral head. Thus, like these authors, 23 we believe that measuring the percentage of subluxation is essential in the preoperative planning of shoulder arthroplasty, and that such variability in the values of this parameter between the manual and automated methods should be taken into consideration, since the percentage of posterior head subluxation is a radiographic criterion that influences the surgical technique to be used and the selection of implants and manual measurements of this parameter are underestimated by the manual method.

As a limitation of the present study, we used a single automated preoperative planning program. We understand that these programs present a variability of measurements among them, since they use different anatomical parameters to format the images.

## Conclusion

The manual method is effective in measuring the glenoid version and inclination when performed by experienced surgeons, and the vault method is more accurate than the Friedman et al. 12 method in patients with glenoids with greater deformities. However, the measurement of the percentage of posterior subluxation of the humeral head presents a great discrepancy between the manual and automated methods. Such differences underscore the need for further studies that aim to assess the impact of such differences on the outcome of procedures, since several uncertainties exist regarding the accuracy of manual and automated methods and the selection and correct positioning of the implants.
