# Supplementary material for: Comparison of Manual Two-dimensional and Automated Three-dimensional Methods of Assessing Shoulder Joint Morphology through Computed Tomography Images
Source: Rev Bras Ortop (Sao Paulo). 2024 Jul 8;59(4):e590–8. doi: 10.1055/s-0044-1786821 (PMC11374392; doi:10.1055/s-0044-1786821)
Supplement: Related texts

# Research article: Comparação de métodos bidimensionais manuais e tridimensionais automatizados de avaliação da morfologia da articulação do ombro por imagens de tomografia computadorizada

**Keywords:** artroplastia, substituição do ombro, tomografia computadorizada por raios X

## Abstract

**Objetivo** Avaliar a concordância interobservador na medida dos parâmetros anatômicos do ombro utilizando métodos manuais de tomografia computadorizada (TC) bidimensional (2D) não formatada no plano da escápula e compará-los à medida automatizada obtida com o programa Blueprint (Wright Medical, Memphis, TN, Estados Unidos), que utiliza imagens tridimensionais (3D) reconstruídas.

**Métodos** Este é um estudo transversal com a utilização de imagens de TC 2D de 38 pacientes com diferentes diagnósticos. Os parâmetros anatômicos foram medidos por métodos manuais - versão, método de Friedman et al., e método do vault; inclinação, por método de Maurer et al., e subluxação do ombro segundo Walch et al. As mensurações foram realizadas por cinco cirurgiões qualificados e independentes, e comparados aos parâmetros obtidos pelo programa automatizado Blueprint.

**Resultados** Foram observadas diferenças significativas entre a versão de medida manual obtida pelo método de Friedman et al. e a versão automatizada. Os valores médios de inclinação não apresentaram diferenças estatisticamente significativas entre os métodos. A média observada de subluxação teve diferenças significativas entre as análises realizadas pelo método automatizado e pelos cirurgiões.

**Conclusão** As medidas manuais de versão e inclinação da glenoide realizadas por cirurgiões experientes são eficazes, e o método da abóbada é superior ao de Friedman et al. na análise de deformidades glenoidais graves.

## Introdução

A correção das deformidades articulares da glenoide é essencial para a realização da artroplastia do ombro, e pode influenciar diretamente o resultado funcional e a sobrevida do implante. 1 Portanto, o planejamento pré-operatório é essencial para o sucesso da artroplastia total do ombro. 1 2 3 4

Tradicionalmente, a avaliação da morfologia da glenoide é realizada por meio de medidas angulares em imagens bidimensionais (2D) de tomografia computadorizada (TC), que demonstraram baixa precisão e concordância inter e intraobservador. 5 O uso de imagens tridimensionais de TC (3D) para atenuar as imprecisões atribuídas ao 2D 6 7 não se mostrou eficaz, pois a correta reconstrução do plano da escápula requer reformatação e processamento manual ou semiautomático das imagens 2D. 6 7

Assim, o surgimento de imagens tomográficas 3D associadas ao desenvolvimento de programas de computador automatizados tem como objetivo controlar as limitações e imprecisões dos métodos manuais e a reformatação dos cortes tomográficos no plano escapular. 8 9 10 11

Portanto, o objetivo deste estudo foi avaliar a concordância interobservador da mensuração dos parâmetros anatômicos do ombro utilizando métodos manuais de TC 2D sem formatação no plano da escápula e compará-los à medida automatizada que utiliza imagens 3D reconstruídas.

## Materiais e Métodos

## Seleção de Casos

Após a aprovação pelo Comitê de Ética em Pesquisa institucional (n° 35243920.4.0000.5273), o estudo avaliou as imagens tomográficas da articulação do ombro de pacientes de ambos os sexos, maiores de 18 anos, com diagnóstico de osteoartrite.

Ao todo, 38 TCs foram selecionadas aleatoriamente dentre exames realizados em pacientes da instituição de janeiro de 2015 a dezembro de 2019. Todos os exames de TC foram realizados no mesmo equipamento (modelo Brilliance, Philips, Amsterdã, Holanda), com 64 canais e o paciente em decúbito dorsal.

Os critérios de inclusão foram TCs com cortes triplanar, coronal, sagital e axial, espessura mínima de corte de 1 mm, visualização de toda a escápula e processamento pelo programa automatizado de planejamento cirúrgico escolhido para o experimento. Foram excluídas imagens de pacientes submetidos a cirurgia prévia no ombro e que apresentavam artefatos nas imagens tomográficas, como presença de implante metálico ou outras alterações anatômicas que pudessem afetar o processamento segmentar pelo programa automatizado.

## Medida Manual

As imagens tomográficas 2D não formatadas no plano da escápula foram utilizadas para a medida manual da versão segundo os métodos de Friedman et al. 12 ( Fig. 1 ) e de vault, descrito por Matsumara et al. 13 ( Fig. 2 ), e de inclinaç ão da glenoide pelo método de Maurer et al. 14 ( Fig. 3 ). Além disso, também medimos o percentual de subluxaç ão da cabeça do úmero de acordo com Walch et al. 4 ( Fig. 4 ).

Essas medidas foram realizadas de forma independente por cinco ortopedistas especializados em cirurgia do ombro, utilizando o programa RadiAnt DICOM Viewer (Medixant, Poznan, Polônia). 15 Os observadores foram instruídos uniformemente, conforme descrito a seguir, para a padronização das medidas. Todos os observadores desconheciam os resultados uns dos outros.

**Fig. 1:** Método de Friedman et al. 12 para a avaliação da versão glenoidal. Foi utilizado o quarto corte distal ao último corte, em que foi visualizada a ponta do processo coracoide no plano axial. A versão glenoidal foi determinada pelo ângulo da seguinte forma: ( **A** ) linha de Friedman et al., 12 ( **B** ) linha glenoidal e ( **C** ) linha perpendicular ao eixo escapular. O valor positivo do ângulo de versão da glenoide foi interpretado como anteversão, ao passo que o valor negativo foi interpretado como retroversão.

**Fig. 2:** Método de *vault* para a medida de versão, que foi definida no corte axial como um triângulo composto pelas paredes anterior e posterior do colo da escápula e pela superfície articular da glenoide. O ângulo é determinado pelas seguintes linhas: ( **A** ) eixo de *vault* , ( **B** ) linha glenoidal e ( **C** ) linha perpendicular ao eixo de *vault* .

**Fig. 3:** Método de Maurer et al. 14 para a medida da inclinação da glenoide, que foi feita no plano coronal oblíquo da tomografia computadorizada, e o corte que capturou de forma melhor o assoalho da fossa supraespinhal foi selecionado. ( **A** ) Linha tangente às bordas anterior e posterior da glenoide, ( **B** ) linha da fossa supraespinhal e ( **C** ) linha perpendicular à linha B determinando a inclinação da glenoide.

**Fig. 4:** Método de Walch et al. 4 para a determinação da subluxação da cabeça do úmero. ( **A** ) Linha tangente às bordas anterior e posterior da glenoide, ( **B** ) linha perpendicular à face da glenoide em seu ponto médio, ( **C** ) linha paralela à linha A, dividindo o terço médio da cabeça do úmero, e ( **D** ) parte da cabeça do úmero posterior ao centro da glenoide. Índice de subluxação = D/C.

## Medida Automatizada

A medida automatizada utilizou imagens de TC no formato Comunicação de Imagens Digitais em Medicina (Digital Imaging and Communications in Medicine, DICOM, em inglês) processadas pelo programa Blueprint 9 (Wright Medical, Memphis, TN, Estados Unidos). O programa realiza um processo automático de segmentação, determina os planos da escápula e da glenoide e, em seguida, determina as medidas, fornecendo os valores de versão e inclinação, além do percentual de subluxação da cabeça do úmero ( Fig. 5 ).

**Fig. 5:** Tela inicial do programa Blueprint com os valores da versão e da inclinação da glenoide e da subluxação da cabeça do úmero.

## Análise Estatística

O coeficiente de correlação interclasse (CCI) foi calculado para determinar a variabilidade das medidas manuais entre os cirurgiões.

Na estatística descritiva, os dados foram apresentados como médias, desvios padrão, valor máximo e valor mínimo. A comparação das medidas obtidas pelos diferentes métodos de avaliação da versão usou o teste de Friedman et al., 12 seguido do pós-teste de Dunn. A comparação entre as medidas de inclinação e subluxação pelos métodos manual e automatizado foi realizada por meio do teste de Wilcoxon. Os casos foram categorizados de acordo com a gravidade da versão e da inclinação, utilizando intervalos de 0 a 10° e > 10° quando indicado. Esses intervalos foram escolhidos com base em estudos que mostraram que a versão normal da glenoide é próxima de 0°, às vezes com leve anteversão, mas, com maior frequência, retroversão com valores tipicamente abaixo de 10°, 16 e que o ângulo de inclinação intrínseco normal da glenoide geralmente é de 0 a 10°. 17 Todas as análises foram realizadas no programa IBM SPSS Statistics for Windows (IBM Corp., Armonk, NY, Estados Unidos), versão 21.0.

## Resultados

O estudo incluiu 38 casos, 17 dos quais receberam o diagnóstico de artropatia do manguito e 21, de osteoartrite.

O CCI das medidas manuais está descrito na Tabela 1 . Os valores médios de versão obtidos pelos métodos de Friedman et al., 12 de *vault* e automatizado são apresentados na Tabela 2 . O método de *vault* foi similar ao método automatizado ( *p* > 0,99), ao passo que o de Friedman et al. 12 tendeu a subestimar essa medida tanto em relação ao método automatizado quanto ao método de *vault* ( *p* = 0,003) ( Tabela 2 ).

**Tabela 1:** Coeficientes de correlação interclasse (CCIs) de versão segundo os métodos de Friedman et al. 12 e da abóbada, inclinação e subluxação

Variáveis	CCI	Intervalo de confiança inferior	Intervalo de confiança superior
** Versão segundo o método de Friedman et al. 12**	0,847	0,751	0,913
**Versão segundo o método da abóbada**	0,845	0,744	0,931
**Inclinação**	0,890	0,821	0,938
**Subluxação**	0,840	0,712	0,917

**Tabela 2:** Medidas de versão

	Blueprint	Friedman et al. 12	Vault
**Versão (média ± desvio padrão)**	10,45° ± 7,1° ^a^	8,07 ± 6,1° ^a, b^	10,42 ± 6,2° ^b^
**Diferença média** **(intervalo de confiança de 95%)**	–	2,37° (0,79–3,9°)	0,023 (−1,8–1,9°)
**Diferença máxima**		11°	13,64°

Notas: ^a^ *p* = 0,0059, teste de Friedman et al., 12 pós-teste de Dunn; ^b^ *p* = 0,0005, teste de Friedman et al., 12 pós-teste de Dunn.

As diferenças entre as medidas da versão feitas pelo Blueprint e pelo método de Friedman et al. 12 foram menores do que 5° em 23 casos (60,5%), entre 5 e 10° em 14 casos (36,8%) e maiores do que 10° em 1 caso (2,6%). Tais proporções foram semelhantes às encontradas na comparação entre o Blueprint e o método de *vault* : 22 (57,8%), 13 (34,2%) e 3 casos (7,8%), respectivamente ( *p* = 0,57) ( Tabela 3 ).

**Tabela 3:** Número de casos em que a média obtida pelas medidas por métodos manuais diferiu das medidas automatizadas, estratificadas por graus de diferença

	Friedman et al. 12	Vault
**Diferença < 5° em relação ao programa Blueprint: n (%)**	23 (60,5%)	21 (57,8%)
**Diferença entre 5 e 10° em relação ao programa Blueprint: n (%)**	14 (36,8%)	13 (34,2%)
**Diferença > 10° em relação ao programa Blueprint: n (%)**	1 (2,6%)	3 (7,8%)

Notas: Teste do qui-quadrado; *p* = 0,57.

A inclinação não apresentou diferença estatística entre os métodos automatizado e manual (11,85° ± 9,8° *versus* 11,24° ± 5,44°; *p* = 0,377) ( Tabela 4 ). As medidas de subluxação resultaram em valores maiores no método automatizado em comparação ao manual (60,08° ± 14,72° *versus* 48,47° ± 7,67°; *p* < 0,0001) ( Tabela 4 ).

**Tabela 4:** Medidas de inclinação e subluxação

	Blueprint	Manual	*p*
**Inclinação (média ± desvio padrão)**	11,85° ± 9,8°	11,24° ± 5,44°	0,377
**Subluxação (média ± desvio padrão)**	60,08° ± 14,72°	48,47° ± 7,67°	< 0,0001

Nota: Teste de Wilcoxon.

Os casos foram categorizados de acordo com a inclinação da glenoide dentro variação anatômica deste parâmetro ( de 10 a þ10° ). Nos casos dentro da normalidade, o método de *vault* gerou valores significativamente maiores do que os do método automatizado ( *p* = 0,04) e do método de Friedman et al. 12 ( *p* = 0,0004). Curiosamente, nos casos com versão superior a 10°, a versão média obtida com o método automatizado foi significativamente superior àquela determinada pelo método de Friedman et al. 12 ( *p* = 0,001), mas semelhante à obtida com o método de *vault* ( *p* = 0,13). Os valores médios de subluxação determinados pelo método automatizado foram significativamente maiores do que aqueles observados com o método manual em ambos os subgrupos avaliados ( Tabela 5 ).

**Tabela 5:** Comparação entre a versão média, a inclinação e a subluxação pelos métodos automatizado e manual e categorizada pela versão medida pelo método automatizado

Versão (°)		Parâmetro		Valor de *p*
**Versão (°): média ± desvio padrão**				
	Blueprint	Friedman et al. 12	Vault	
0–10 (n = 21)	5,6 ± 2,8 ^a^	5,4 ± 4,0 ^b^	7,7 ± 3,9 ^a, b^	0,01*
> 10 (n = 17)	16,3 ± 6,5 ^c^	11,3 ± 6,9 ^c^	13,7 ± 7,0	0,0061*
**Inclinação (°): média ± desvio padrão**				
	Blueprint		Manual	
0–10 (n = 21)	11,5 ± 11,5		10,9 ± 6,0	0,7 ^#^
> 10 (n = 17)	10,8 ± 5,0		11,4 ± 4,8	0,5 ^#^
**Subluxação (°): média ± desvio padrão**				
	Blueprint		Manual	
0–10 (n = 21)	51,3 ± 10,4		45,7 ± 7,2	0,005 ^#^
>10 (n = 17)	69,8 ± 12,5		52,0 ± 7,1	< 0,0001 ^#^

Notas: *Análise de variância ( *analysis of variance* , ANOVA, em inglês) unidirecional; ^a^ *p* = 0,04; ^b^ *p* = 0,0004; ^c^ *p* = 0,001; teste de comparações múltiplas de Tukey; ^#^ teste *t* .

Os casos foram então categorizados considerando-se a inclinação medida pelo método automatizado. Nos casos com inclinação dentro da normalidade, a versão média obtida pelo método de *vault* foi significativamente superior àquela determinada pelo método de Friedman et al. 12 ( *p* = 0,1), sem diferenças entre o método automatizado e o manual. Nos casos com inclinação superior a 10°, a versão média foi significativamente subestimada pelo método de Friedman et al. 12 em comparação ao método automatizado ( *p* = 0,002) e ao método da abóbada ( *p* = 0,007).

Nos casos com inclinação dentro da normalidade, esse parâmetro foi superestimado pelo método manual em comparação ao automatizado ( *p* = 0,001). Em relação à subluxação, os valores médios obtidos pelo método automatizado foram significativamente maiores do que aqueles observados com o método manual em ambos os subgrupos avaliados ( Tabela 6 ).

**Tabela 6:** Comparação entre a média da inclinação pelos métodos automatizado e manual e categorizada pela gravidade da inclinação medida pelo método automatizado

Inclinação (°)		Parâmetro		Valor de *p*
**Versão (°): média ± desvio padrão**				
	Blueprint	Friedman et al. 12	Vault	
0–10 (n = 21)	8,9 ± 6,4	8,3 ± 6,2 ^a^	10,9 ± 7,3 ^a^	0,03*
> 10 (n = 17)	12,3 ± 7,8 ^b^	7,7 ± 6,3 ^b,c^	9,7 ± 4,8 ^c^	0,003*
**Inclinação (°): média ± desvio padrão**				
	Blueprint		Manual	
0–10 (n = 21)	5,9 ± 2,9		8,7 ± 3,8	0,001 ^#^
> 10 (n = 17)	17,6 ± 9,9		14,0 ± 5,8	0,1 ^#^
**Subluxação (°): média ± desvio padrão**				
	Blueprint		Manual	
0–10 (n = 21)	60,33 ± 15,0		49,9 ± 6,8	0,0001 ^#^
> 10 (n =17)	58,7 ± 14,5		46,8 ± 8,6	0,0008 ^#^

Notas: *Análise de variância ( *analysis of variance* , ANOVA, em inglês) unidirecional; ^a^ *p* = 0,01; ^b^ *p* = 0,002; ^c^ *p* = 0;007; teste de comparações múltiplas de Tukey; ^#^ teste *t* .

## Discussão

O sucesso da artroplastia do ombro depende do posicionamento adequado do implante. 1 A habilidade do cirurgião em identificar alterações morfológicas na glenoide, principalmente quanto à versão e à inclinação, é de extrema importância, pois evita deslocamentos que comprometem a sobrevida do procedimento. 2 4 7

Entretanto, os métodos manuais 2D para a definição da morfologia da glenoide apresentam limitações e imprecisões devido à dificuldade de segmentação das imagens tomográficas no plano anatômico da escápula. As discrepâncias motivaram o desenvolvimento de programas automatizados para o planejamento pré-operatório, cuja utilização tem se tornado cada vez mais frequente para melhorar o entendimento da anatomia, o posicionamento e a fixação dos componentes protéticos. 8 9 Porém, seu uso rotineiro ainda não ocorre na prática da maioria dos cirurgiões que realizam artroplastias de ombro. 8 18 19 20 Além disso, existem controvérsias quanto à precisão desses métodos e ao posicionamento ideal dos implantes. 21 22 23 24 25

Na prática clínica de países de rendas baixa e média, imagens de TC 2D não formatadas no plano da escápula são amplamente utilizadas, o que, apesar de ser uma limitação, é uma realidade. No presente estudo, as medidas manuais da versão, tanto pelos métodos de Friedman et al. 12 quanto de *vault* , da inclinação e da subluxação apresentaram bom CCI. Curiosamente, outros estudos 5 6 9 10 demonstraram que a versão da glenoide medida em imagens de TC 2D apresenta significativa variabilidade interobservador devido à variação na rotação coronal e sagital da escápula em relação à posição do paciente na mesa de exame. No entanto, o presente estudo obteve um bom CCI, o que atribuímos ao fato de todos os avaliadores serem cirurgiões de ombro com mais de cinco anos de formação e experiência em artroplastias desta articulação.

No presente estudo, a versão média da glenoide medida pelo método automatizado foi semelhante à obtida pelo método de *vault* . Porém, a versão medida pelo método de Friedman foi inferior à obtida pelos métodos automatizado e de *vault* . Ainda assim, com base nas comparações das versões, apenas 1 caso apresentou diferença superior a 10° entre o método de Friedman et al. 12 e o automatizado. Portanto, demonstramos que o método de Friedman et al. 12 é menos preciso do que o de *vault* ao considerarmos o método automatizado como padrão. Em 2014, Matsumara et al. 13 compararam a medida da versão pelos métodos de Friedman et al. 12 e de *vault* e, da mesma forma, afirmaram que ambos os métodos apresentam boa concordância interobservador, mas que o método de *vault* facilita a medida, pois não depende das variações anatômicas do corpo da escápula ou de sua inclusão no exame. 11 13 15 23

Notavelmente, ao usar o método automatizado como padrão nos casos em que a versão estava dentro da normalidade, não observamos diferenças significativas entre o método automatizado e o de Friedman et al.; 12 no entanto, nos casos com versão fora da normalidade (> 10°), a versão média obtida pelo método de Friedman et al. 12 foi significativamente inferior à observada com o método automatizado. Em conjunto, esses resultados permitem concluir que a utilização da medida de versão pelo método automatizado é mais importante nos casos em que as deformidades na superfície da glenoide são mais graves, podendo influenciar a prática clínica tanto na correção da versão quanto no correto posicionamento do implante. Esse achado é compatível com o encontrado na literatura, em que Chalmers et al. 26 demonstraram que a medida da versão baseada em imagens tomográficas é superior 26 27 nas deformidades B2 segundo a classificação morfológica proposta por Walch et al. 4

Em um estudo recente, Reid et al. 28 observaram resultados semelhantes aos nossos, e mostraram que a medida da versão em TCs 2D pelo método de Friedman et al. 12 apresenta altas taxas de correlação intra e interclasse. Por outro lado, este estudo 28 também encontrou diferenças significativas entre medidas utilizando métodos manuais e automatizados. Porém, quando os casos foram categorizados de acordo com a gravidade da versão, os autores 28 não encontraram diferenças entre os métodos nos diversos subgrupos avaliados, e sugeriram que a magnitude da versão não influencia as diferenças entre os métodos. Este resultado contrasta com os achados do presente estudo, em que constatamos que o método manual subestimou a inclinação da glenoide em comparação ao método automatizado nos casos com versão superior a 10°. As diferentes faixas de versões utilizadas para estratificar os casos e os diferentes diagnósticos incluídos em nossa série podem explicar essas discrepâncias.

A inclinação glenoidal apresentou valores semelhantes aos da avaliação por métodos manuais ou automatizados. Em medidas de inclinação baseadas em TCs formatadas, Iannotti et al. 21 observaram que não houve diferenças em relação às medidas obtidas pelo método automatizado. Em relação às imagens não formatadas, a diferença foi expressiva. Em 94% dos casos, a diferença entre a inclinação das imagens não formatadas e as formatadas foi superior a 5°, novamente um valor com significância na prática clínica. 21 Com os casos categorizados de acordo com a gravidade da inclinação, o presente estudo detectou diferenças na medida dos casos brandos (inclinação entre 0–10°), em que o método manual gerou uma média significativamente superior à obtida pelo método automatizado ( *p* = 0,001). Em um estudo de 2020, Choi et al. 29 compararam a inclinação medida por métodos automatizados e 2D e descobriram que o método 3D gerou medidas significativamente inferiores às obtidas com o método 2D. Embora os autores 29 não tenham realizado análise de subgrupo, os dados avaliados apresentaram valores médios de versão e inclinação compatíveis com os casos incluídos no subgrupo de casos brandos do presente estudo.

Em relação à subluxação, houve diferenças significativas entre os métodos manual e automatizado. Outros estudos 30 já relataram erro intrínseco à medida da subluxação pelo método de Walch et al. 4 O índice de subluxação, descrito por Walch et al., 4 é medido em corte transversal da TC. O corpo escapular apresenta inclinação em relação ao corpo do paciente, em que os cortes transversais da TC estão alinhados, o que leva a erros nas medidas da TC 2D. 5 10 11 26 27 30 Jacxsens et al. 30 concluíram que medidas baseadas em ombros reconstruídos em 3D parecem ser mais adequadas, pois reconstroem a anatomia óssea independentemente da orientação do paciente no momento do exame; assim, essas medidas podem ser mais confiáveis do que as obtidas em 2D, que são subestimadas.

Um achado interessante do presente estudo foi o de que as diferenças entre as medidas de subluxação obtidas pelos métodos automatizado e manual tenderam a aumentar com a gravidade da versão, o que sugere que, quanto maior o ângulo de versão, mais o método manual subestima a medida da subluxação quando o padrão é o método automatizado. Mais estudos são necessários para avaliar o impacto clínico de tal achado, uma vez que pode influenciar diretamente o planejamento cirúrgico.

Chalmers et al. 26 avaliaram os valores de versão e inclinação da glenoide e de subluxação da cabeça do úmero por meio de TC corrigida e não corrigida, e compararam essas medidas aos valores fornecidos pelo Blueprint. Eles 26 concluíram que a orientação dos cortes das TCs formatadas no plano da escápula levou à diminuição das medidas de retroversão em comparação às TCs não formatadas. Por outro lado, os autores 26 verificaram que não há diferenças entre as TCs não formatadas e os valores dos programas automatizados. Em 48% dos casos, a diferença entre as versões não corrigida e corrigida foi superior a 5°, o que é considerado significativo na prática clínica. 21 Esses achados são semelhantes aos resultados obtidos em nossa série.

Shukla et al. 23 utilizaram o mesmo programa automatizado do presente estudo e encontraram resultados semelhantes entre os métodos manual e automatizado nas medidas de versão e inclinação, mas com diferença importante nos percentuais de subluxação da cabeça do úmero. Logo, assim como esses autores, 23 acreditamos que a mensuração do percentual de subluxação é essencial no planejamento pré-operatório da artroplastia do ombro, e que tal variabilidade nos valores desse parâmetro entre os métodos manual e automatizado deve ser levada em consideração, pois o percentual de subluxação posterior da cabeça é um critério radiográfico que influencia a técnica cirúrgica a ser utilizada, e a seleção dos implantes e as medidas deste parâmetro são subestimadas pelo método manual.

Como limitação do presente estudo, utilizamos somente um programa automatizado de planejamento pré-operatório. Entendemos que esses programas apresentam variabilidade de medidas entre si, pois empregam diferentes parâmetros anatômicos para a formatação das imagens.

## Conclusão

O método manual é eficaz nas medidas da versão e da inclinação da glenoide quando realizado por cirurgiões experientes, e o método de *vault* é mais preciso do que o de Friedman et al. 12 em pacientes com glenoides com deformidades maiores. Porém, a medida do percentual de subluxação posterior da cabeça do úmero apresenta grande discrepância entre os métodos manual e automatizado. Tais diferenças reforçam a necessidade de mais estudos que visem avaliar o impacto de tais diferenças no resultado dos procedimentos, uma vez que existem diversas incertezas quanto à precisão dos métodos manuais e automatizados e à seleção e ao posicionamento correto dos implantes.
